# Identifying coevolving loci using interspecific genetic correlations

**DOI:** 10.1002/ece3.3107

**Published:** 2017-07-28

**Authors:** Scott L. Nuismer, Christina E. Jenkins, Mark F. Dybdahl

**Affiliations:** ^1^ Department of Biological Sciences University of Idaho Moscow ID USA; ^2^ School of Biological Sciences Washington State University Pullman WA USA

**Keywords:** association study, coevolution, genetics of adaptation, genome scans, infection genetics, landscape genetics, local adaptation, resistance, SNP genotyping, virulence

## Abstract

Evaluating the importance of coevolution for a wide range of evolutionary questions, such as the role parasites play in the evolution of sexual reproduction, requires that we understand the genetic basis of coevolutionary interactions. Despite its importance, little progress has been made identifying the genetic basis of coevolution, largely because we lack tools designed specifically for this purpose. Instead, coevolutionary studies are often forced to re‐purpose single species techniques. Here, we propose a novel approach for identifying the genes mediating locally adapted coevolutionary interactions that relies on spatial correlations between genetic marker frequencies in the interacting species. Using individual‐based multi‐locus simulations, we quantify the performance of our approach across a range of coevolutionary genetic models. Our results show that when one species is strongly locally adapted to the other and a sufficient number of populations can be sampled, our approach accurately identifies functionally coupled host and parasite genes. Although not a panacea, the approach we outline here could help to focus the search for coevolving genes in a wide variety of well‐studied systems for which substantial local adaptation has been demonstrated.

## 1 INTRODUCTION

Host–parasite coevolution has the potential to drive many evolutionary transitions: from sexual to asexual reproduction (Hamilton, 1980; Jaenike, 1978; Lively, 1987), from haploidy to diploidy (Nuismer & Otto, 2004), and from selfing to outcrossing (Agrawal & Lively, 2001). What has become increasingly apparent, however, is that coevolution's role in these evolutionary transitions depends on the genetic details of the interaction (Agrawal & Otto, 2006; Lively, 2010; Otto & Nuismer, 2004). Thus, in order to make concrete predictions about the likely long‐term evolutionary consequences of coevolution, we need to better understand the genetic details of interactions between hosts and parasites.

Despite long‐standing interest, identifying the genes involved in coevolution has proven difficult. The primary reason for this is that understanding the genetic basis of coevolution requires identifying the suites of genes in host and parasite that interact to determine the outcome of the interaction (Ebert, 2008; Heath & Nuismer, 2014; Thrall, Barrett, Dodds, & Burdon, 2016). Thus, in contrast to studies of single species where it may be sufficient to identify genes that influence a phenotype of interest, studies of coevolution must identify genes in both host and parasite that interact with one another to produce a composite phenotype such as resistance or virulence. As a consequence, established approaches for identifying genes influencing single species phenotypes, such as genetic association tests, can identify genes important in each species, but never which genes in one species interact with which genes in the other. A result of this difficulty is that we have a substantial collection of studies identifying candidate genes for resistance in hosts (e.g., Atlija, Arranz, Martinez‐Valladares, & Gutierrez‐Gil, 2016; Benavides et al., 2015; Kim, Sonstegard, da Silva, Gasbarre, & Van Tassell, 2015; Kover & Caicedo, 2001; Kover, Wolf, Kunkel, & Cheverud, 2005; Magwire et al., 2012; Redmond et al., 2015; Wilfert & Schmid‐Hempel, 2008) and infectivity in parasites (e.g., Molina‐Cruz et al., 2013; Scanlan, Hall, Lopez‐Pascua, & Buckling, 2011) , but virtually no studies that identify evolutionarily coupled genes in host and parasite.

A novel approach for identifying evolutionarily coupled genes in interacting species is suggested by coevolutionary theory (Morgan, Gandon, & Buckling, 2005; Nuismer & Gandon, 2008). This theory demonstrates that if a reciprocal cross‐infection experiment reveals local adaptation of one species to the other, it must be the results of spatial covariation between the frequencies of functionally interacting genes in host and parasite (Nuismer & Gandon, 2008). Thus, for the wide range of host–parasite interactions where parasite local adaptation has been demonstrated (Greischar & Koskella, 2007; Hoeksema & Forde, 2008; Lively, 1989), it should be possible to identify the genes involved in coevolution by identifying genetic markers in each species that covary with one another across populations. Thus, as with approaches that seek to identify the genetic basis of local adaptation by searching for statistical associations between genotype frequencies and environmental variables (Coop, Witonsky, Rienzo, & Pritchard, 2010; Gunther & Coop, 2013; Hancock et al., 2011; Hoban et al., 2016; Joost et al., 2007), our approach seeks to identify genes involved in coevolution by searching for statistical associations between genotype frequencies in the interacting species.

Here, we formalize the use of statistical associations between host and parasite gene frequencies as a tool for identifying regions of host and parasite genomes involved in coevolution. We begin by developing the theoretical background that underpins the approach. Next, we lay out a step‐by‐step implementation of the approach for a scenario where frequencies of genetic markers have been estimated for host and parasite populations. Finally, we use multilocus individual‐based simulations to evaluate the performance of our approach for a wide range of coevolutionary scenarios and parameter values.

## 2 OVERVIEW OF APPROACH

### 2.1 Theoretical background

Studies of host–parasite coevolution frequently estimate the extent to which parasites or hosts are adapted to their local antagonist populations using a reciprocal cross‐infection experiment (Greischar & Koskella, 2007; Hoeksema & Forde, 2008; Nuismer & Gandon, 2008). Often such studies rely on sampling host and parasite individuals from *N* populations and confronting them with one another in a fully reciprocal design. The result is an *N *× *N* matrix with entries corresponding to the average infection rate, *P*_*i,j*_, of parasites drawn from population *i* when confronted with hosts drawn from population *j*. Parasite local adaptation, L, can then be calculated as the difference between expected infection rate when confronted with local hosts and expected infection rate when confronted with all hosts, irrespective of location:

(1) $$L=\sum_{i=1}^{N}P_{i,i}-\sum_{i=1}^{N}\sum_{j=1}^{N}P_{i,j}.$$

Using a very general model, Nuismer and Gandon (2008) showed that this expression can be re‐written in terms of the spatial covariance between host and parasite genotype frequencies:

(2) $$L=\sum_{i=1}^{n_{P}}\sum_{j=1}^{n_{H}}\alpha_{i,j}\text{Cov}\left[X_{i},Y_{j}\right]$$

where $\alpha_{i,j}$ is the infection rate of a parasite with genotype $X_{i}$ when confronted with a host of genotype *Y*_*j*_, $\text{Cov}\left[X_{i},Y_{j}\right]$ is the covariance between the frequency of parasite genotype *i* and host genotype *j* over the populations included in the cross‐infection experiment, and *n*_*k*_ is the number of genotypes within species *k* that influence the probability of infection. This result demonstrates that in systems where the parasite is locally adapted, frequencies of genotypes that result in infection must have a positive covariance across populations. In contrast, in systems where the host is locally adapted, frequencies of genotypes that result in infection must have a negative covariance across populations. Thus, in systems where local adaptation of one species to the other has been observed through a reciprocal cross‐infection experiment, it must be the result of a spatial covariance between frequencies of genotypes that influence infection. This suggests that identifying host and parasite genes with frequencies that covary across space provides a potentially useful tool for identifying the genetic basis of coevolution and local adaptation.

### 2.2 Implementation

The theoretical results outlined above suggest that when local adaptation is observed, searching for coevolving regions of the genome by looking for genes with spatially covarying frequencies may be a profitable approach. We emphasize that the approach we propose here is only likely to be effective, and the results interpretable, when local adaptation has first been estimated experimentally using a reciprocal cross‐infection experiment. Assuming local adaptation has been demonstrated experimentally, implementing this approach is straightforward in principle and can be accomplished through the following steps. First, identify a set of candidate genes or markers (e.g., SNP's) within each of the interacting species. Second, calculate the frequencies of these genes or markers within each of the *N* populations for which local adaptation has been estimated. Third, calculate the spatial covariance between the frequencies of each host and parasite gene or marker. The result is a matrix of covariances between the frequency, $p_{H,i}$, of host genotype/marker *i*, and the frequency, $p_{P,j}$, of parasite genotype/marker *j*:

(3) $$C=\left[\begin{array}{ccc} \text{Cov}\left[p_{H,1},p_{P,1}\right] & \cdots & \text{Cov}\left[p_{H,1},p_{P,n_{P}}\right]\\ \vdots & \ddots & \vdots \\ \text{Cov}\left[p_{H,n_{H}},p_{P,1}\right] & \cdots & \text{Cov}\left[p_{H,n_{H}},p_{P,n_{P}}\right] \end{array}\right]$$

where the matrix has a number of rows equal to the number of host genotypes/markers and a number of columns equal to the number of parasite genotypes/markers. Screening this potentially enormous matrix for statistical associations between host and parasite genotype/marker frequencies can be simplified by transforming the covariances into correlations using the standard statistical formula:

(4) $$\rho_{i,j}=\frac{\text{Cov}\left[p_{H,i},p_{P,j}\right]}{\sigma_{p_{H,i}}\sigma_{p_{P,i}}}$$

where $\sigma_{p_{H,i}}$ and $\sigma_{p_{P,i}}$ are the standard deviations of marker/genotype frequencies $p_{H,i}$ and $p_{P,j}$ across the *N* study populations. Using (4) to transform the covariance matrix (3) results in a matrix of spatial correlations for all possible pairs of host and parasite markers/genotypes:

(5) $$\rho =\left[\begin{array}{ccc} \rho_{1,1} & \cdots & \rho_{n_{P},1}\\ \vdots & \ddots & \vdots \\ \rho_{1,n_{H}} & \cdots & \rho_{n_{P},n_{H}} \end{array}\right]$$

With this correlation matrix in hand, it is a simple matter to quickly screen combinations of host and parasite markers/genotypes for statistical significance by calculating a test statistic, $t_{i,j}$, for each correlation:

(6) $$t_{i,j}=\;\frac{\rho_{i,j}\sqrt{N-2}}{1-\rho_{i,j}^{2}}$$

where *N* is the total number of populations in the study. Finally, compare the value of the test statistic *t*_*i,j*_ to the critical value of *t* drawn from the Student's *t*‐distribution with *N *− 2 degrees of freedom and the desired significance level, α, for each correlation. This statistical approach is strictly correct only in cases where populations evolve independently of one another (i.e., no gene flow, no historical population genetic structure) and loci also evolve independently of one another. If these conditions do not hold, correlations may not follow a *t*‐distribution and the degrees of freedom will certainly be overestimated. In the subsequent section, we use individual‐based simulations to grossly violate these key assumptions of our statistical approach and to evaluate the consequences of these violations for both type I and type II error rates. In the discussion, we introduce alternative statistical approaches and avenues for future statistical development that may prove to be more efficient. The result of the screening procedure we propose is a list of matched pairs of host and parasite markers/genotypes that correlate significantly with one another across space. The stringency of this screen, and the number of false positives, can be adjusted using different values of α.

## 3 INDIVIDUAL‐BASED SIMULATIONS

The previous section lays out a straightforward methodology for identifying candidate pairs of host and parasite markers/genes responsible for observed patterns of interspecific local adaptation and potentially also involved in the coevolutionary process. To evaluate how well this method is likely to work in practice, we tested it using genetically explicit individual‐based simulations. Simulations followed a metapopulation of host and parasite individuals consisting of *N* populations, each of which contained η haploid host and parasite individuals. The genomes of host and parasite individuals consisted of *n*_*H*_ and *n*_*P*_ diallelic loci, of which a randomly selected subset were assumed to be involved in the coevolutionary interaction. Loci not involved in the coevolutionary interaction had no impact on fitness and thus evolved neutrally in response to random genetic drift, gene flow, mutation, and indirect selection. These neutral loci provide an important control and allow us to explore whether processes other than coevolution (e.g., population structure) can confound our approach. Simulations proceeded by following individuals through a life‐cycle consisting of: (1) species interactions, (2) reproduction, (3) mutation, and (4) migration. Each stage of this life cycle is detailed below.

### 3.1 Species interactions

**Figure 1:**
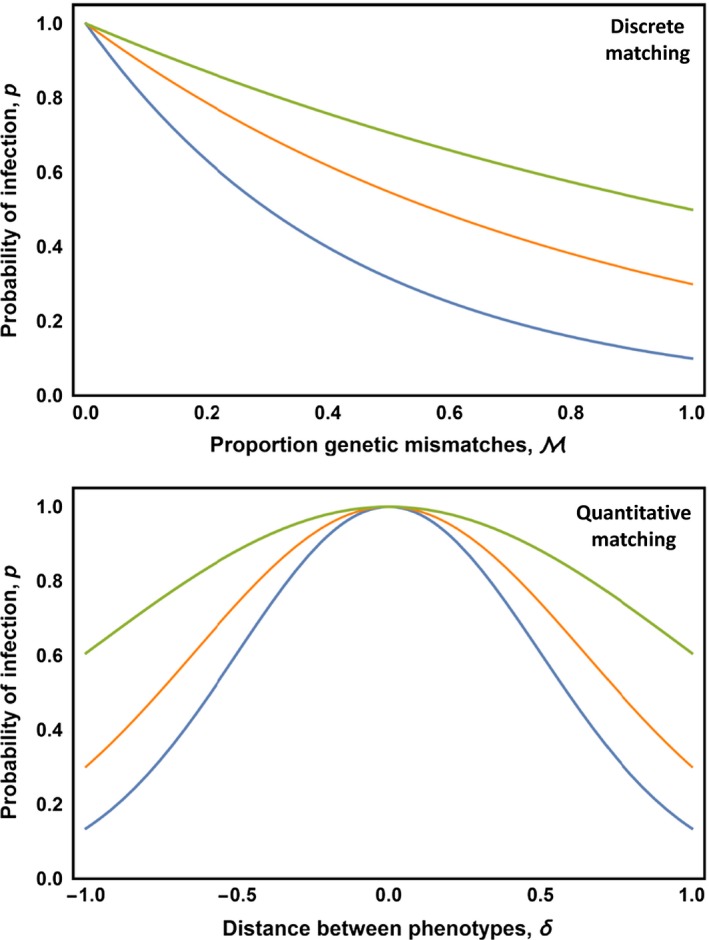
The relationship between the proportion of genetic mismatches at coevolving loci, M, and the probability of infection for the discrete matching model (top panel) and the relationship between scaled phenotypic distance, δ, and the probability of infection for the quantitative matching model (bottom panel). For the discrete matching model, the green line corresponds to β=0.5, the orange line to β=0.7, and the blue line to β=0.9. For the quantitative matching model, the green line corresponds to β=0.5, the orange line to β=1.2, and the blue line to β=2.0

Individual hosts and parasites were assumed to encounter one another at random within each population, with each host individual encountering exactly one parasite individual. Random encounters between host and parasite individuals resulted in either infection or resistance, with the probability of infection determined by one of two coevolutionary models. In the first model, which we refer to as the “discrete matching” model, the probability of infection depends on the proportion of the coevolving loci that carry matching alleles in host and parasite. Specifically, this model assumes that the probability of parasite infection is greatest when host and parasite individuals have precisely matched genotypes and decreases as the proportion of mismatched loci, M, increases such that:

(7) $$P\left(X_{h},X_{p}\right)={\left(1-\beta \right)}^{M}$$

where the parameter β determines how sensitive the probability of infection is to the degree of genetic mismatching between host and parasite (Figure 1). As the parameter β approaches 1, this model converges on a classical matching alleles model of coevolution where parasites can infect only those hosts with perfectly matching genotypes.

The second coevolutionary model we consider, which we refer to as the “quantitative matching” model, assumes the probability of infection depends on the difference, δ, between a quantitative trait in the host, $z_{h}$, and a quantitative trait in the parasite, $z_{p}$ (Figure 1). Specifically, we assume the probability of infection is greatest when host and parasite phenotypes match and declines as the distance between host and parasite phenotypes increases such that:

(8) $$P\left(z_{h},z_{p}\right)=exp\left[-\beta \delta^{2}\right]$$

where $\delta =z_{h}-z_{p}$. Within this expression, the host and parasite phenotypes *z*_*h*_ and *z*_*p*_ are determined by summing the number of “1” alleles each individual carries at the subset of loci involved in coevolution. The parameter β determines how sensitive the probability of infection is to the difference between the individual's phenotypes. Within simulations, phenotypes of host and parasite were scaled to always lie between zero and one.

For each random encounter between host and parasite, the fitness of the host individual is equal to one minus the product of the infection probability and the virulence of infection, *s*:

(9a) $$W_{h}=1-sP$$

and the fitness of the parasite individual is equal to the probability of successful infection:

(9b) $$W_{p}=P$$

After calculating fitness, a random number was drawn from a uniform distribution on [0,1] for each individual, and if that number was greater than the fitness calculated by (9) the individual was eliminated from the population.

### 3.2 Reproduction, mutation, and migration

Individual hosts and parasites that successfully survived species interactions were allowed to reproduce sexually. Mating occurred by selecting a random pair of haploid parents and producing a new haploid offspring. Offspring was produced following standard rules of Mendelian inheritance with recombination occurring between adjacent loci *i* and *j* at rates $r_{H,i,j}$ and $r_{P,i,j}$ in host and parasite, respectively. Random mating continued until a new population of zygotes was created of a size equal to the original population size, η. After reproduction, each genome experienced a mutation with probabilities $\mu_{H}$ and $\mu_{P}$ in host and parasite, respectively. Mutation was symmetrical and converted the current allele at a randomly selected locus to its alternative form. Finally, individuals migrated at random among neighboring populations with probabilities $m_{H}$ and $m_{P}$ in host and parasite, respectively. Thus, migration followed a linear stepping stone model and was symmetric— if an individual from one population migrates to another population, then a replacement must migrate back to the migrant's starting population. Populations located at the two ends of the linear sequence of populations experienced migration at half the rate of interior populations (because they have only a single neighboring population).

## 4 QUANTIFYING PERFORMANCE

After simulating coevolution for 500 generations, local adaptation was calculated by conducting a simulated reciprocal cross‐infection experiment and applying Equation (1). Spatial correlations were then calculated for all possible pairs of host and parasite loci to generate the correlation matrix described by (5). Statistically significant correlations were then identified using (6) and a range of significance levels spanning α=0.001 and α=0.020. Type I error rates were quantified by summing the number of matrix entries that were falsely identified as coevolving (statistically significant correlations between neutral loci or between coevolving loci that were not functionally paired) and dividing by the total possible number of type I errors. Type II error rates were calculated by summing the number of matrix entries between coevolving loci that were not identified (no statistically significant correlation between functionally paired loci) and dividing by the total possible number of type II errors. In cases where multiple loci were involved, we considered cases where only a subset of the loci involved were identified as errors. Thus, our estimates of type II errors are conservative. Simulations were run for scenarios where coevolution depended on 1, 2, or 3 loci and for metapopulations consisting of 30, 40, and 50 populations. For each combination of coevolving loci and metapopulation size, remaining parameters were assigned as described in Table 1, and simulations were run repeatedly until at least 30 replicate simulations were accumulated for each of the following strengths of local adaptation: minimal (0<L≤0.10), weak (0.10<L≤0.15), moderate (0.15<L≤0.20), and strong (0.20<L). These values of local adaptation were chosen to span the range observed in empirical studies of naturally occurring host–parasite interactions (Table 2).

**Table 1:** Parameter values used in simulations and their biological interpretations

Parameter	Meaning	Values
*n*_*H*_	Host background genome size	Fixed at 100
*n*_*P*_	Parasite background genome size	Fixed at 100
*m*_*H*_	Host movement rate	Drawn at random from a uniform distribution on [0, 0.01]
*m*_*P*_	Parasite movement rate	Drawn at random from a uniform distribution on [0, 0.01]
β	Sensitivity of infection to host and parasite genotypes	Drawn at random from a uniform distribution on [0.8, 1.0] for the discrete matching model and on [2.0, 4.0] for the quantitative matching model
S	Virulence of infection	Drawn at random from a uniform distribution on [0.6, 0.9]
$\eta_{H}$	Local host population size	Drawn at random from a uniform distribution on [150, 300]
$\eta_{P}$	Local parasite population size	Drawn at random from a uniform distribution on [150, 300]
$\mu_{H}$	Host genome wide mutation rate	Drawn at random from a uniform distribution on [0.01, 0.05]
$\mu_{P}$	Parasite genome wide mutation rate	Drawn at random from a uniform distribution on [0.01, 0.05]
$r_{H,i,j}$	Host recombination rate between adjacent loci *i* and *j*	Drawn at random from a uniform distribution on [0, 0.5]
$r_{P,i,j}$	Parasite recombination rate between adjacent loci *i* and *j*	Drawn at random from a uniform distribution on [0, 0.5]

**Table 2:** Estimates of local adaptation from reciprocal cross‐infection studies. We reviewed studies of local adaptation and identified those where a reciprocal cross‐infection study was performed in the laboratory, allowing local adaptation to be calculated using Equation (1) and the resulting value compared directly to simulation results. In some cases, we selected fully reciprocal combinations from larger studies and thereby excluded populations that were not reciprocally exposed. Bold entries are those for which local adaptation is sufficiently large for our technique to be useful

Estimated magnitude (averaged across replicates)	Species	Reference
0.058	*Microbotryum violaceum* *Silene latifolia*	(Kaltz, Gandon, Michalakis, & Shykoff, 1999)
0.108	*Melampsora amygdalina* *Salix triandra*	(Niemi, Wennström, Hjältén, Waldmann, & Ericson, 2006)
0.110	*Schistocephalus solidus* *Gasterosteus aculeatus*	(Weber et al., 2017)
**0.188**	***Microphallus*** **sp.** ***Potamopyrgus antipodarum***	**(Lively,** 1989 **)**
**0.252**	***Microphallus*** **sp.** ***Potamopyrgus antipodarum***	**(Lively & Dybdahl,** 2000 **)**
**0.236**	***Protopolystoma*** **spp.** ***Xenopus laevis***	**(Jackson & Tinsley,** 2005 **)**
0.103	Plasmodium spp. Parus major	(Jenkins, Delhaye, & Christe, 2015)

**Figure 2:**
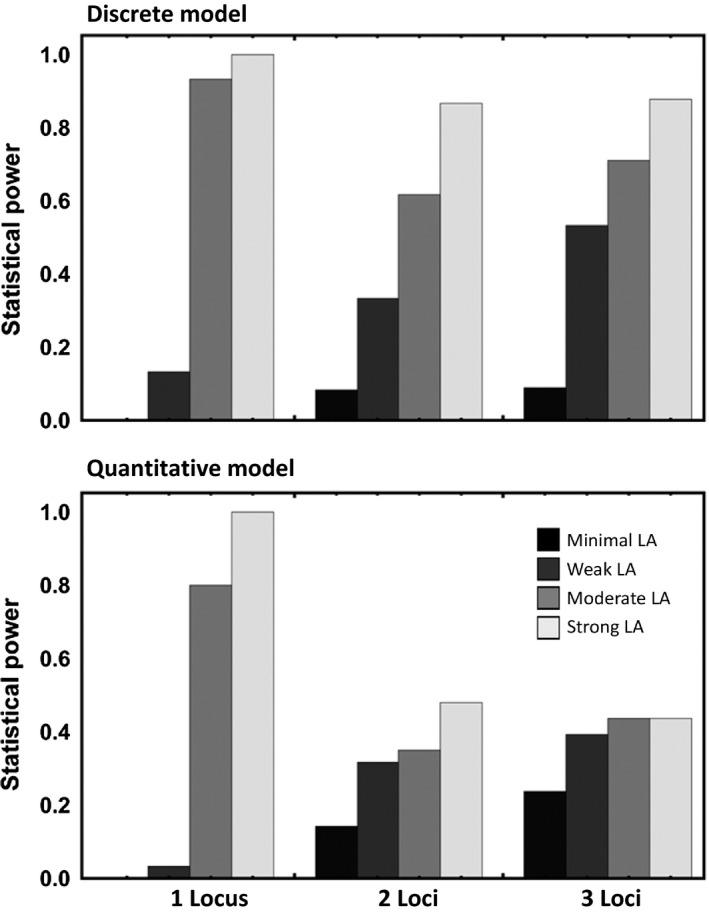
Statistical power as a function of the strength of local adaptation (bar shading) and the number of loci (bar groups) for the discrete matching model (top panel) and the quantitative matching model (bottom panel). Values of local adaptation were 0.0≤L≤0.10 (black bars), 0.10<L≤0.15 (dark gray bars), 0.15<L≤0.20 (gray bars), and 0.20<L (light gray bars). Data results from simulations where forty populations were sampled, and the significance level, α, was set to 0.01

Simulation results demonstrated that scanning host and parasite genomes for markers/genotypes with frequencies that covary across space can be an effective tool for identifying loci involved in coevolutionary interactions. For instance, when local adaptation was strong $\left(\left|L\right|>0.20\right)$, coevolution depended on only a single locus, and 40 or more populations were sampled, our method identified the coevolving pair of loci in 100% of simulations (Figure 2). The method performed almost as well for cases of more modest local adaptation $\left(0.15<\left|L\right|\leq 0.20\right)$, successfully identifying the coevolving locus in 93.3% of cases for the discrete matching model and 80.0% of cases for the quantitative matching model when only a single locus was involved (Figure 2). In contrast to the promising results observed in cases where local adaptation was substantial, simulations suggest the statistical power of our method drops precipitously when local adaptation is weak (magnitude less than 15%) (Figure 2). This result is not surprising, of course, because coevolutionary theory predicts spatial associations between genotype frequencies should be proportional to the magnitude of local adaptation (Nuismer & Gandon, 2008). In addition to weak local adaptation, sampling from a relatively small number of populations (≤20) greatly reduces the power of our approach (results not shown). As a general rule of thumb, unless local adaptation is exceptionally strong (e.g., $\left|L\right|>0.30$), our approach is only likely to be useful when it is possible to include twenty or more populations in the study.

**Figure 3:**
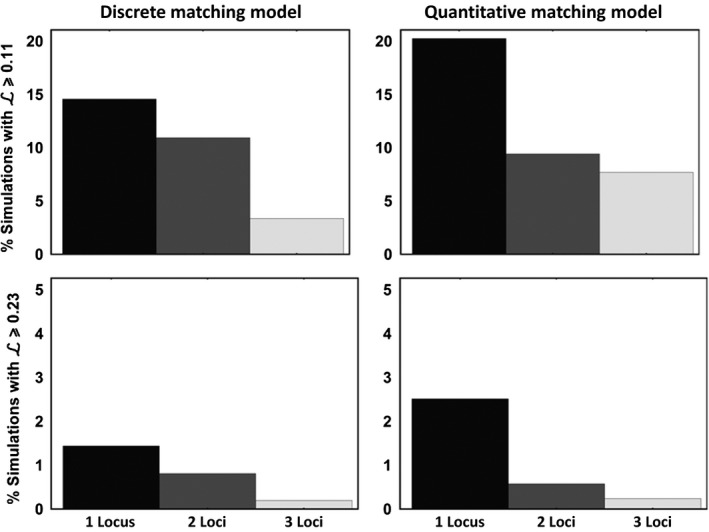
The percentage of simulations yielding a magnitude of local adaptation exceeding a threshold value for the discrete matching model (left hand column) and the quantitative matching model (right hand column). In the top row, the threshold value of local adaptation is modest (L=0.11) corresponding to the middle ground of the estimates for local adaptation reported in Table 2. In the second row, the threshold of local adaptation was more extreme (L=0.23), corresponding to the largest estimates of local adaptation reported in Table 2. In all cases, simulations demonstrate that large values of local adaptation are more likely to result when coevolution is mediated by a small number of genes with large effect

Although our simulation results demonstrate that our approach is quite effective at identifying coevolving genes when only a single locus is involved, the power of the approach declines with increasing numbers of coevolving loci in some cases. Specifically, for the quantitative matching model, statistical power drops when more than a single locus is involved in the coevolutionary process (Figure 2). In contrast, for the discrete matching model, increasing the number of coevolving loci has a much less substantial impact on statistical power (Figure 2). This difference in behavior arises because loci in the discrete matching model interact epistatically and are thus not interchangeable, whereas loci in the quantitative matching model interact additively and are thus interchangeable. Consequently, if substantial local adaptation is observed in the discrete matching model, it must be that the frequencies of all loci involved in coevolution covary across space; in contrast, for the quantitative matching model, substantial local adaptation can occur when only a subset of allele frequencies covary across space. Although the drop in statistical power with increasing numbers of coevolving loci is, in principle, a problem, the results of our simulations strongly suggest that when substantial local adaptation is observed in a host–parasite interaction, it is most likely to be the result of coevolution mediated by a small number of genes with major effects on the probability of infection (Figure 3).

**Figure 4:**
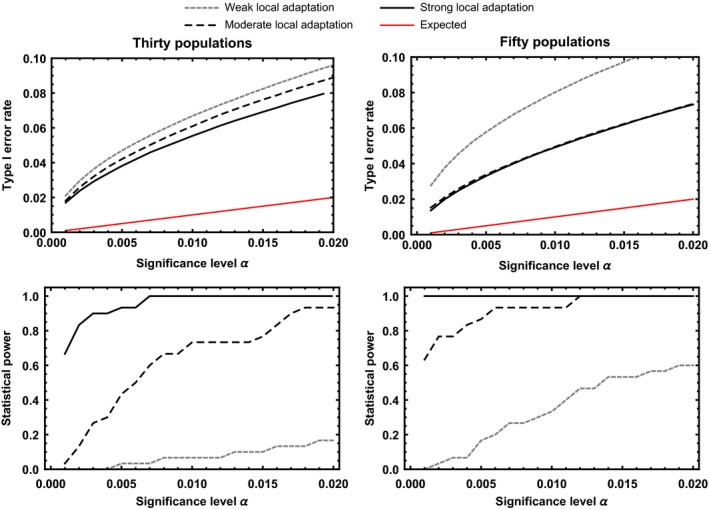
Type I error rates and statistical power for the discrete matching model as a function of the significance level, α, for three different levels of local adaptation and cases where thirty populations are sampled (left hand column) or fifty populations are sampled (right hand column). The gray‐dotted line shows cases where local adaptation is modest (0.1≤L≤0.15) and in such cases, statistical power is low unless a large number of populations is sampled. The black dashed line shows cases where local adaptation is more substantial (0.15≤L≤0.20) and in such cases, statistical power is substantially improved. The solid black line shows cases where local adaptation is strong (0.20≤L), and in such cases, statistical power is very good, even when only thirty populations are sampled. The red line shows the expected type I error rate given the significance level, α

**Figure 5:**
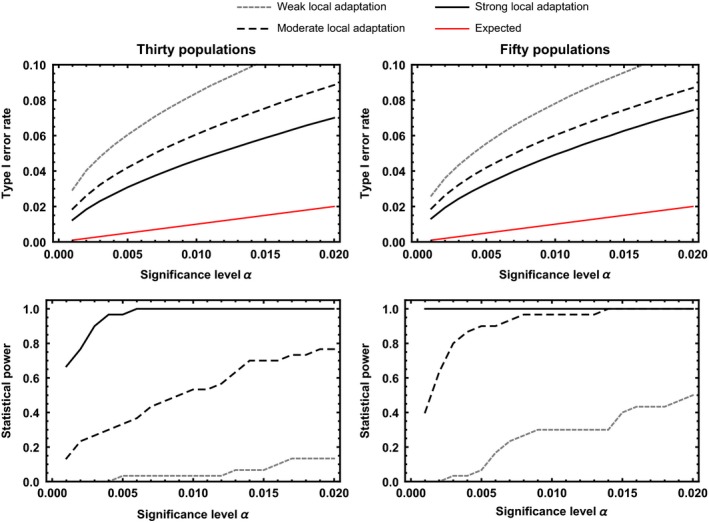
Type I error rates and statistical power for the quantitative matching model as a function of the significance level, α, for three different levels of local adaptation and cases where thirty populations are sampled (left hand column) or fifty populations are sampled (right hand column). The gray‐dotted line shows cases where local adaptation is modest (0.1≤L≤0.15), and in such cases, statistical power is low unless a large number of populations is sampled. The black‐dashed line shows cases where local adaptation is more substantial (0.15≤L≤0.20), and in such cases, statistical power is substantially improved. The solid black line shows cases where local adaptation is strong (0.20≤L), and in such cases, statistical power is very good, even when only thirty populations are sampled. The red line shows the expected type I error rate given the significance level, α

**Figure 6:**
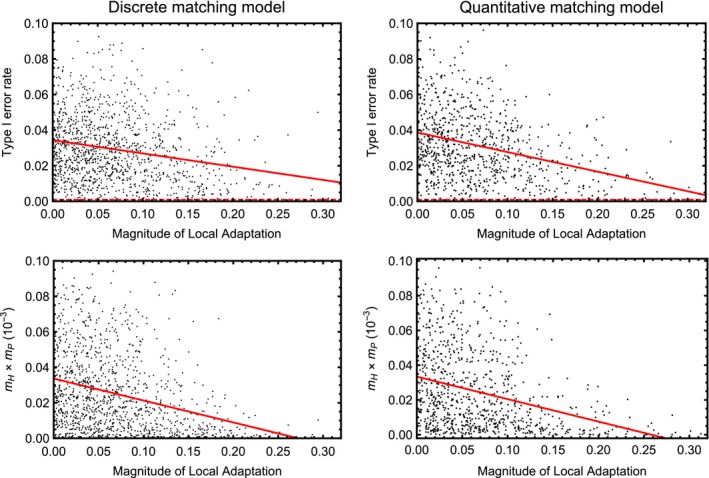
Type I error rates and the product of host and parasite migration rates plotted as a function of the magnitude of local adaptation for the discrete matching model (left hand column) and the quantitative matching model (right hand column). Data results from simulations where coevolution was mediated by a single genetic locus, but patterns are similar for larger numbers of loci. The red line is the best fit of a negative exponential model to the data and is included only as an aid to visualization. The significance level, α, was set to 0.001 in these simulations (shown by the dashed red line), and as local adaptation increases, the median type I error rate converges on this value

Taken together, the results of coevolutionary simulations suggest that our approach can be an effective tool for identifying the major genes contributing to local adaptation in coevolving interactions between hosts and parasites. At the same time, of course, our approach also falsely identifies neutral loci as coevolving in some cases. Quantifying the type I error rate of our approach using simulations demonstrates that, on average, the type I error rate is inflated, with the degree of inflation inversely proportional to the magnitude of local adaptation (Figures 4 and 5). The primary reason the type I error rate becomes inflated is that our statistical test assumes populations are independent; an assumption that is clearly violated in the presence of substantial gene flow. We further investigated the relationship between the magnitude of local adaptation and the type I error rate by plotting the type I error rate as a function of the magnitude of local adaptation (Figure 6; top row). The results of this investigation demonstrate that type I error rates are greatly inflated when local adaptation is weak, but fall as the magnitude of local adaptation increases. In fact, as the magnitude of local adaptation becomes very large, the median value of the type I error rate converges on the significance level, α, set by the investigator. The primary reason the type I error rate falls as the magnitude of local adaptation increases is simply that very strong local adaptation occurs only when rates of gene flow become very low and populations evolve more or less independently of one another (Figure 6; bottom row). Thus, as long as local adaptation has been experimentally demonstrated to be strong, a priori, type I error rates are only moderately inflated above the user‐defined significance level.

## 5 DISCUSSION

We have developed a new methodology for identifying the genes mediating coevolutionary interactions. Our method capitalizes on well‐established theory demonstrating that local adaptation of one species to another must be the result of spatial associations between frequencies of coevolving genes in the interacting species (Morgan et al., 2005; Nuismer & Gandon, 2008). This new methodology represents a significant advance over existing techniques because it has the potential to identify functionally paired genes across species rather than genes adapting independently in each species. Extensive simulation testing of our method demonstrates that it performs well if local adaptation is strong ($\left|L\right|>0.15$) and marker frequencies can be estimated from thirty or more populations. In contrast, if local adaptation is weak or marker frequencies cannot be estimated from at least twenty populations, the statistical power of our approach is poor and the false discovery rate can become extremely high.

From a practical standpoint, our simulation results suggest our methodology will be limited to a subset of empirical systems where coevolution produces strong local adaptation. Unfortunately, this means our approach cannot be applied to systems where coevolution does not cause strong local adaptation, such as arms races mediated by quantitative traits (e.g., Brodie, Ridenhour, & Brodie, 2002; Ridenhour & Nuismer, 2007). It also means that our approach should not be applied in cases where local adaptation has not been first demonstrated experimentally. Although these limitations narrow the scope of application, some important and well‐studied systems do meet the requirements of our approach (Table 2; bold entries). Furthermore, given the wide range of host–parasite systems where strong local adaptation has been observed (but which we did not include in our table because the units were not directly comparable to those of our simulations), opportunities for applying our approach should be substantial (Greischar & Koskella, 2007; Hoeksema & Forde, 2008).

Although our approach provides a new method for identifying interacting genes in coevolving species, it shares many of the limitations of existing approaches based on genotype–environment associations (Hoban et al., 2016). For instance, our approach works best when coevolution and local adaptation depend on a small number of loci with large phenotypic effects (Korte & Farlow, 2013; Rockman, 2012). Our approach also requires that genomic coverage is sufficiently dense for markers to lie within, or in close proximity to, the genes involved in the coevolutionary interaction. An additional potential complication could arise if both host and parasite are jointly adapted to a common feature of the abiotic environment. For all of these reasons, it is important to recognize that our approach provides only a coarse initial screen for identifying candidate pairs of loci involved in coevolution. Sifting through the resulting collection of candidate coevolving gene pairs to nail down the genetic basis of coevolution will require the use of a wide range of existing techniques and ultimately experimental verification (Cantor, Lange, & Sinsheimer, 2010). In addition to these standard limitations of genetic association studies, our approach requires the accurate estimation of genetic marker frequencies within a pair of interacting species across a relatively large number of populations (>20). Although a daunting challenge in many systems, rapidly decreasing genotyping costs suggest this barrier will continue to decline, making our approach financially feasible in a wide range of natural systems.

As we have outlined it here, our approach relies upon a very simple statistical test that identifies unusually large correlations by comparing them to the distribution expected under a null model where all populations and loci are independent of one another. The strength of this approach is that it is quick and easy to implement and performs well in cases of strong local adaptation. A significant weakness of this approach, however, is that it can lead to inflated type I error rates when gene flow among populations is significant and local adaptation more moderate. As has been previously demonstrated for single‐species genotype–environment association studies, inflation of type I error rates becomes increasingly acute as isolation by distance increases (Lotterhos & Whitlock, 2014). As a consequence, the technique we present here should work best in situations where isolation by distance is weak or absent, as is likely to be the case in systems where gene flow approximates an island model.

Future work could significantly improve on the approach we develop here by developing methods that correct for the impact of population structure. For instance, following research on single‐species studies, it may be possible to generate null distributions of interspecific correlations using the simulations we developed here coupled with a known demographic history or estimated patterns of movement among populations (e.g., Beaumont & Nichols, 1996; Eckert et al., 2010; Excoffier, Hofer, & Foll, 2009). An obvious weakness of this approach, however, is that it relies on an accurate knowledge of demographic history or patterns of movement (Hoban et al., 2016; Lotterhos & Whitlock, 2014). Alternatively, it may be possible to correct for the impact of neutral population structure by estimating the covariance structure among marker frequencies from the data itself (e.g., Bonhomme et al., 2010; Gunther & Coop, 2013). A strength of this approach is that it does not require knowledge of demographic history or estimates of patterns of movement; a weakness is that loci involved in coevolution may be used to correct for neutral population structure, thus reducing statistical power (Hoban et al., 2016). Developing these additional tools, and testing them using genetically explicit coevolutionary simulations has the potential to greatly expand the number of systems in which our approach can be applied.

In summary, the approach we develop here introduces a novel methodology for identifying the genetic basis of coevolving interactions in cases where local adaptation has been estimated a priori and shown to be strong. Our approach provides significant advantages over existing techniques, the most important of which is the ability to identify not just individual genes in each of the interacting species, but also the interactions between these genes across species. Although not a panacea, the approach we outline here could help to focus the search for coevolving genes in a wide variety of well‐studied systems for which local adaptation has been demonstrated. More importantly, by demonstrating that interspecific genetic correlations carry the signature of coevolution, our results pave the way for future approaches that correct for population structure and remove key limitations of the statistically crude approach presented here.

## CONFLICT OF INTEREST

None declared.
